# Assessing the educational impact of cognitive level of MCQ and SEQ on learning approaches of dental students

**DOI:** 10.12669/pjms.37.2.3475

**Published:** 2021

**Authors:** Mahwish Arooj, Khadijah Mukhtar, Rehan Ahmed Khan, Tayyaba Azhar

**Affiliations:** 1 Mahwish Arooj, MBBS, MME, M. Phil, PHD Physiology. Professor of Physiology and Director, DME University College of Medicine and Dentistry, Lahore, Pakistan; 2 Khadijah Mukhtar, BDS, MME Assistant Professor, DME University College of Medicine and Dentistry, Lahore, Pakistan; 3 Rehan Ahmed Khan, MBBS, FCPS, FRCS, MHPE Professor of Surgery, Assistant Dean Medical Education, Riphah International University; 4 Tayyaba Azhar. MBBS, MME Assistant Professor, DME University College of Medicine and Dentistry, Lahore, Pakistan

**Keywords:** Learning approaches, Study Process Questionnaire (SPQ), MCQ’s and SEQ’s

## Abstract

**Objectives:**

MCQ’s and SEQ’s are the most widely used assessment tool in dental colleges across Pakistan. This study explores the impact of assessment tool: MCQ’s and SEQ’s on learning approach of dental students and also identifies correlation between these assessment tools and deep & surface learning approaches in integrated and discipline based curriculum.

**Methods:**

A quantitative co-relational study was conducted in 2018 on 2nd and 4th year dental students. A pre-validated “Revised Study Process Questionnaire” was used. Spearman’s rho correlation coefficient and Wilcoxon signed ranks test were applied to determine the relationship between learning approaches and assessment tools. Internal consistency was calculated by Cronbach’s alpha.

**Results:**

Ninety six students out of one hundred and fifty completed the questionnaire. Correlation test showed that surface approach correlates significantly with MCQ’s (0.73) while no co-relation exists with SEQ’s (-0.14) in our study. Deep approach has a strong and significant correlation with SEQ’s (0.80) as compared to MCQ’s (0.056).

**Conclusion:**

Assessment tool has an impact on learning approaches used by the students. It was concluded that students used to prefer deep learning approach while preparing for SEQ’s as they were designed at higher cognitive level, whereas, they preferred surface approach while preparing for MCQ’s as they were developed at low cognitive order.

## INTRODUCTION

Assessment has a direct influence on the learning approach of the students. It plays a major role in defining the content, depth, strategy and approach that students use in order to pass the examination.^1^ Deep and surface approach are two predominant approaches used by the students.^2^ Students using surface approach focus only on rote memorization in order to pass the examination. Therefore, they fail to develop the actual concepts of the subject. Whereas, students who adopt deep approach develops an understanding of the concepts and relate new learning with the previous knowledge, resulting in the retention of information and developing critical thinking skills.^3^

Learning approach is a dynamic characteristic influenced by various factors like student’s self-motivation, learning environment perception about assessment, assessment methods as well as assessment tools.^4^ One of the reasons that students adopt surface learning is when the assessment tool only assesses lower level of cognition. Contrary to this, they adopt a deep approach when the assessment is focused on assessing deeper understanding of the subject.^5^,^6^ Multiple Choice Questions (MCQs) and Short Essay Questions (SEQs) are two commonly used assessment tools in medical and dental colleges^7^ and can assess lower to higher order cognition based on their construct.^8^

Various studies have been conducted to find the relationship between learning approaches and assessment type by using Study Process Questionnaire (SPQ).^9^,^10^ However, to the best of our knowledge, no study was found in the literature which showed the educational impact of most widely used assessment tool: SEQ’s and MCQ’s on learning approaches. This study has been conducted to assess the impact of MCQ’s and SEQ’s on learning approaches used by dental students and to identify the correlation of assessment tools (MCQ’s Vs SEQ’s) with surface and deep learning approaches in integrated and discipline based curriculum.

### Hypothesis of the study

Null hypothesis of this study was, ‘students do not use surface approach to study in MCQ (exam designed at low cognition level) and deep approach for SEQ (exam designed at higher cognitive level)’. Two alternate hypotheses established were: (1) ‘students primarily use surface approach to study for MCQ and deep approach for SEQ’ and (2) ‘students primarily use deep approach to study in MCQ and surface approach for SEQ’.

## METHODS

It is a quantitative co-relational study conducted in six months on students of Bachelor’s in Dental Surgery (BDS) at University College of Dentistry, the University of Lahore, approved by the Ethical Review Board (Ref: ERC/5/5/17, Dated: May 02, 2019) of the University. Ninety-six students from Second year and fourth year BDS students were included in the study. Sample size was calculated with 80% power of test and 5% level of significance by taking expected 81% of total population of dental students. Due to recent implementation of integrated curriculum in the dental college second year students are taught through integrated modular curriculum while fourth year students are following traditional curriculum. In both these classes, the theory examination comprised of MCQ and SEQ, while the skill examination is conducted through laboratory practical, OSPE, short case, long case, viva and logbook. MCQ’s are mostly designed at the recall, understanding and application level whereas SEQ’s are developed at understanding and problem-solving level. This is because it is more feasible for the faculty to develop a smaller number of assessment items at higher cognitive level.

The learning approach of the students while preparing for MCQ and SEQ examination were assessed by using a validated, 20 item ‘Revised Study Process Questionnaire’, developed by Biggs.^11^ To check the consistency and student’s understanding, the questionnaire was piloted with 20 randomly selected students. All statements were contextualized with respect to SEQ and MCQ examination preparation for final professionals which comprised of 64 MCQs and 12 SEQs. After piloting, the questionnaire was distributed among the second year and fourth year students during free time at favorable setting. The objectives of the study were explained to the students and were assured about their confidentiality. Written consent was taken from the participants after which they filled the questionnaire. Two separate questionnaires to assess the educational impact for MCQ & SEQ were used and responses were recorded on two different days to avoid any confusion.

Wilcoxon signed ranks test was applied to compare the learning approaches with MCQ and SEQ. P-value of < 0.10 was considered significant for this study.^12^ Spearman’s rho correlation coefficient was applied to see their relationship. Reliability was determined by Cronbach’s alpha.

## RESULTS

In 2^nd^ year and 4^th^ year BDS, total 96 students completed the questionnaire. There were 33.3% (32) male and 66.6% (64) female students who filled the questionnaire and participated in this study. The reliability (internal consistency) on both samples was calculated. Reliability of questionnaire second year students using surface approach for MCQs was 0.69 and for SEQ’s was 0.70 while the reliability for deep approach for MCQs was 0.67 and for SEQs was 0.71(Table-I).

**Table-I:** Reliability Test.

*Class*	*Variable*	*Items*	*MCQs*	*SEQs*	*Cronbach Alpha*	*Number of students*
Second year	Surface	10	0.69	0.71	0.69	52
	Deep	10	0.67	0.71	0.66	52
Fourth year	Surface	10	0.68	0.7	0.67	44
	Deep	10	0.84	0.74	0.84	44

Moreover, the reliability of questionnaire for fourth year students using surface approach for MCQs is 0.68 and for SEQ’s is 0.70 while the reliability of SPQ for deep approach for MCQs is 0.84 and for SEQs is 0.74 as shown in (Table-I).

Spearman Correlational test was applied to comprehend the linear relationship between two variables (Deep and Surface, MCQ and SEQ). In both classes surface approach correlates with MCQ’s strongly than SEQ’s while deep approach correlates strongly with SEQ’s as compared to MCQ’s (Table-II).

**Table-II:** Correlational Test.

*Approaches*	*Variable*	*Correlation value*	*P-Value*
Deep	MCQ’s	0.05	0.09
	SEQ’s	0.80	0.4
Surface	MCQ’s	0.73	0.03
	SEQ’s	-0.14	0.46

Hypothesis 1 which proposed that the students used surface approach for MCQ and deep approach for SEQ is accepted as the results showed a significant value for second year (0.05) and fourth year (0.006) (Table-III).

**Table-III:** Hypothesis Testing.

*Class*	*Variable*	*MCQ’s Mean(SD)*	*SEQ’s Mean(SD)*	*Wilcoxon signed ranks test (z)*	*P Value*	*Comment*
Second year	Deep SEQ’s and Surface MCQ’s	2.62 (0.63)	2.85 (0.65)	-1.95	0.05	Hypothesis accepted
	Surface SEQ’s and Deep MCQ’s	2.87 (0.61)	2.70 (0.63)	-1.49	0.14	Hypothesis not accepted
Fourth year	Deep SEQ’s and Surface MCQ’s	2.60 (0.64)	2.96 (0.68)	-2.74	0.006	Hypothesis accepted
	Surface SEQ’s and Deep MCQ’s	2.86 (0.80)	2.60 (0.67)	-1.26	0.21	Hypothesis not accepted

Hypothesis 2 which suggested that the students used surface approach for SEQs and deep approach for MCQs is rejected due to insignificant value for second year (0.14) and fourth year (0.21). Similarly, null hypothesis is also rejected as there is significant association of deep approach with SEQ’s and surface approach with MCQ’s in both classes.

There is no significant difference in adopting deep and surface approaches by the students of 2^nd^ year and 4^th^ year as shown in Table-III. Also, there is no significant difference seen with respect to the learning approaches used by the students in integrated and traditional curriculum.

## DISCUSSION

Current research is centered on the learning approaches used by the students when studying for a particular type of assessment tool (MCQ’s and SEQ’s). The result of this study shows that students used deep approach for the preparation of SEQ’s that were designed at higher cognitive level of application and problem solving and surface approach for MCQ’s that were mainly addressing rote learning and understanding. The main reason for this educational impact is that the MCQ’s used in our study were assessing lower level of cognition and SEQ’s were developed to assess higher level of cognition. Other likely reason responsible for this consequential validity of the assessment items is also that MCQ’s have potential of guessing as compared to SEQ’s.^13^

Furthermore, SEQs in general warrants students to answer the question in detail as compared to the MCQs, hence requiring them to understand and memorize the educational contents so they can be reproduced in the examinations based on SEQs and also includes the fact that the process of essay writing involves analytical and critical thinking skills.^14^,^15^ It is important to note that student can employ both surface and deep learning while preparing for essay questions, but the ratio of students using deep approach for SEQ preparation is significantly higher which is due to the reason that SEQ’s require higher level of cognition and students need to have an in-depth knowledge of the subject to perform well in SEQ’s.^16^

We also found that students significantly preferred surface approach over deep approach while preparing for MCQ’s (as shown in Table-III). The association of surface learning and MCQ examination is well documented in literature but the relation of learning approach with SEQ is not yet explored. Studies conducted by Watkins (1982), Ramsden (1988) and Scoullar (1998) showed that the students used surface learning strategy while preparing for multiple-choice examination.^17^ Another study reported a similar pattern in which the students favored surface approach as compared to the deep approach while preparing for MCQ’s.^4^ Stanger-Hall KF (2000) suggested that multiple-choice examination hinders students’ high cognitive thinking.^18^

According to present study, there is no significant difference in deep and surface approaches adopted by the students of 2^nd^ year and 4^th^ year (Table-III). Similarly, Eun Kyung Mung also did not find significant change in use of approach amongst 1^st^ year and 4^th^ year students. According to him this is because students of 4^th^ year face less fear of failure.^19^ Whereas, Warren Lake in 2015 suggested that students in senior classes were more likely to adopt deep approach of learning.^20^ McDonald F, et al. suggests that students develop deep learning approach over a period of time.^21^ Difference amongst results of these studies may be due to change in sample size, curriculum, discipline and assessment & teaching methodologies in different schools.^22^

The current study also shows that learning approach is not influenced by the type of curriculum. Students studying in both traditional and integrated curriculum used deep approach for SEQ and surface approach for MCQ. The construct of SEQ’s is most likely the reason that students use the deep approach as they are being assessed at a higher level of cognition.^19^ Researchers also suggest that students enrolled in integrated curriculum used both deep and surface approaches equivalently which is in line with the results of our study. This was due to the fact that the teaching in integrated curriculum was multidisciplinary, it was more contextualized which helped the students to relate different concepts and made them active learner. 11

Students are strategic learners when it comes to learning and assessment.^23^ Assessment is one of the major contributors which defines the way students will learn. It is pertinent to use the appropriate assessment tool that will assess higher order of thinking thus motivating the student to develop an in-depth understanding of the subject rather than assessing factual recall of knowledge. This study provides an evidence which can be used by medical and dental colleges to standardize the assessment (MCQ’s & SEQ’s) at high cognition level, which means that both MCQ’s and SEQ’s should be constructed in such a way that the student should use deep approach while preparing for the exam rather than selecting surface learning approach, thus improving the quality of assessment tool. However, there is a need to further explore the other factors which may also influence the learning approach adopted by the students other than examination tool.

### Limitations of the study

It includes lack of standardization of MCQ and SEQ development because they are not formulated at the same level of cognition. This study was conducted with the existing assessment practices used at the institute which did not ensure the uniformity among various assessment tools with respect to level of cognition.

## CONCLUSIONS

Assessment tool has an impact on the learning approaches used by students. It is pertinent to mention that learning approach is also influenced by the construct of the assessment tool. Hence, there is a need to improve its quality in order to ensure deep learning.

### Author’s Contribution:

**MA** **& RAK:** Conceived the idea and designed the study.

**KM & MA:** Collected the data. Responsible for accuracy and integrity of work

**MA, KM, RAK & TA:** Contributed towards data analysis and writing the manuscript and approved the final version.
